# Supplementary material for: Weak ties and the value of social connections for autistic people as revealed during the COVID-19 pandemic
Source: Commun Psychol. 2025 Mar 6;3:36. doi: 10.1038/s44271-025-00208-7 (PMC11883032; doi:10.1038/s44271-025-00208-7)
Supplement: Supplementary file 2 — Supplementary Information [file 44271_2025_208_MOESM2_ESM.pdf]

Supplementary Information

Pellicano & Heyworth: Weak ties and the value of social connections for autistic people as revealed during the COVID-19 pandemic

Supplementary Table 1.

Standards for Reporting Qualitative Research (SRQR) Checklist

Index	Topic	Page(s)
	**Title and Abstract**	
1	Title	1
2	Abstract	2
	**Introduction**	
3	Problem formulation	3-6
4	Purpose or research question	6
	**Methods**	
5	Qualitative approach and research paradigm	11
6	Researcher characteristics and reflexivity	11-12
7	Context	6-7
8	Sampling strategy	7
9	Ethical issues pertaining to human subjects	10
10	Data collection methods	10-11
11	Data collection instruments and technologies	10-11
12	Units of study	7-10
13	Data processing	11
14	Data analysis	11
15	Techniques to enhance trustworthiness	11
	**Results**	
16	Synthesis and interpretation	12-21
17	Links to empirical data	12-21
	**Discussion**	
18	Integration with prior work, implications, transferability, and contribution(s) to the field.	21-26
19	Limitations	27
	**Other**	
20	Conflicts of interest	28
21	Funding	28

Reference:

O'Brien, B. C., Harris, I. B., Beckman, T. J., Reed, D. A., & Cook, D. A. (2014). Standards for reporting qualitative research: a synthesis of recommendations. *Academic Medicine: Journal of the Association of American Medical Colleges*, *89*(9), 1245–1251. https://doi.org/10.1097/ACM.0000000000000388

Supplementary Table 2.

Interview schedules. Participants were asked all primary questions, which they received prior to the interview. Prompt questions helped the interviewer to gain more detailed information.

Participant	Primary questions	Question Prompts
**Young person**	LIVING: Who are you living with at the moment? Do you have any brothers or sisters? Any pets? Have you and your family been staying at home? Tell me about that experience.	What has been DIFFICULT about being asked to stay at home? And what are some of the GOOD things about being asked to stay at home?<br>Have you been able to get out of the house (e.g., going for a walk/to the park/to the shops)? Tell me about those experiences.
	LEARNING: Have you been going to school? If no, have there been classes online and work set by your teacher(s)? Do you like the way you are being taught? Why/why not? Are you doing much work? If yes, is going to school now different to what it was before the coronavirus? If so, how?	Do you need someone to help you with your work? Who is helping you if you need someone? Why do you like/dislike the way you are being taught?<br>What are your favourite subjects? Are they the same favourite subjects as they are when you are at school?<br>What are the GOOD things about being schooled from home? What are some of the NOT-SO-GOOD things about being schooled from home?
	CONNECTING WITH OTHERS: Do you have people you like to spend time or play with? Do you have people you share an interest with? What are their names, and what do you normally like doing together?<br>Have you been able to see them/speak to them during COVID-19?	How have you seen/spoken to them (e.g., face-to-face at home, face-to-face out and about, over video chat, text chat, over the phone, email, online gaming)? What have you enjoyed about that? Have you missed doing other things with them?<br>Have you felt lonely during this time? What do you do when you feel lonely?<br>Do you usually see, speak to, or spend time with your neighbours? Has that changed during COVID-19?
	WELLBEING: Tell me what you know about COVID-19/the coronavirus.<br>Have you had trouble sleeping? Are you liking more routine? Are you stimming more?<br>What makes you feel NOT-SO-GOOD about your life at the moment (during the pandemic)? Can you describe some examples for me?<br>What makes you feel GOOD about your life at the moment? Describe some examples for me.	What do you do to help you feel better when you have trouble sleeping/like more routine/stim more [feel stressed or anxious] (e.g., go for a walk, talk to a friend/parents, do things you enjoy doing, overeat/eat too little, stay in bed longer than usual)?<br>Do you get to have time for your interests/focusing on projects? And time to relax? Has COVID-19 affected those things?
	GETTING HELP: Who do you go to when you need help or feel worried/stressed?	Were you getting any help before COVID-19 happened? Were you seeing a psychologist or a speech therapist or anything like that? Has this changed since

		COVID-19? In what way (e.g., moved to online)? Do you like the way that this has changed?<br><br>Do you feel like you need extra help at the moment? What kind of extra help do you think you need?
	LIFE AFTER COVID-19: It is difficult to know what life after COVID-19 might look like. What things are you worried about? What things are you looking forward to? If you could tell me one positive thing about the COVID-19 experience, what would it be?	
**Autistic adult**	LIVING: Tell me about where you live (e.g., in a house, block of flats). Have you been staying at home? Tell me about that experience.	Are there other people in your household? Tell me about your experience of living together under the COVID-19 restrictions.<br><br>What about pets – do you have any pets?<br><br>What changes to your day-to-day life (including routines) have you had to make? How has that been for you?<br><br>Have you been able to go outside? Tell me about those experiences.<br><br>Are you in a romantic relationship with a person that is not living with you? Have you been able to see them face-to-face? How have you been communicating with them?<br><br>What has been difficult about staying at home? Have there been any positive things about staying at home?
	WORKING/STUDYING: Before COVID-19, where you studying or in employment? What did you do/what were you studying? How has this changed during COVID-19?	[Depending on response to primary question] Are you considered a key worker? Have you been going to work? Tell me about those experiences.<br><br>If working from home, what are some of the advantages of working from home? What are some of the disadvantages?<br><br>Have you been worried about your day-to-day finances?<br><br>If studying, have all of your classes gone online? Have you been able to access these online classes? What are your experiences of learning in this way? Have you received support from your teachers/lecturers/tutors?<br><br>If not working/studying, how have you been filling your days?
	CONNECTING WITH OTHERS: Did you have friends (online/face-to-face) and other people (e.g., support workers, colleagues, extended family) you connected with, prior to COVID-19? How have these interactions changed? If so, in what way?	Who have you had contact with since the COVID-19 restrictions? How has that contact taken place (e.g., face-to-face, over video chat, text chat, over the phone, email, online gaming)?

		Do you engage with the autistic community? How has that been during COVID-19? Have you been more/less engaged?
	WELLBEING: In what ways do you think COVID-19 has affected your physical wellbeing? In what ways do you think COVID-19 has affected your mental wellbeing?	Have your eating/exercise/sleeping patterns changed? In what ways?<br><br>What about time for your passions? And leisure time? Has COVID-19 affected those things?<br><br>In what ways have you been taking care of yourself? Has this been affected by COVID-19?<br><br>Do you have someone you can ask for support if you need (support workers and or friends/family)? Has this been affected by COVID-19?<br><br>Have you noticed any negative changes to your wellbeing? Are you finding it harder than you usually do to motivate yourself to engage in your passions/interests? Are you finding it harder than you usually do to organise yourself? If yes, please could you tell me about this.<br><br>Have you noticed any positive changes to your wellbeing? If yes, please could you tell me about this.
	SUPPORT AND SERVICES: Were you accessing any professional support prior to COVID-19 (e.g., for personal care, psychological therapies etc.)? What kind of support? Are you continuing to receive this support? How has it changed?	Do you have an NDIS plan? Has the plan/funding you receive changed in any way since COVID-19? What about the services you're accessing using NDIS funds – have they changed? In what way(s)?<br><br>[Depending on answer to previous question] Have you applied/were you intending to apply for an NDIS plan prior to COVID-19? Why/why not?<br><br>Many services have moved online. Have you been able to access these services? Have you chosen to access these services? Why/Why not? If yes, have they been useful? If no, how has this affected your physical/mental/emotional health?<br><br>Have you felt you've needed extra support during COVID-19? What kind of extra support might help?<br><br>What has been the most HELPFUL source of support you've received?<br><br>What has been the most UNHELPFUL source of support you're received?
	LIFE AFTER COVID-19: It is difficult to know what life after COVID-19 might look like. What are your concerns for yourself? Your family? And your community (e.g., the Autistic community, your work community)?	

	What things are you optimistic about for yourself? Your family? And your community (e.g., the Autistic community, your work community)?<br>If there was one positive thing about the COVID-19 experience, what would it be?	
