# Supplementary material for: Weak ties and the value of social connections for autistic people as revealed during the COVID-19 pandemic
Source: Commun Psychol. 2025 Mar 6;3:36. doi: 10.1038/s44271-025-00208-7 (PMC11883032; doi:10.1038/s44271-025-00208-7)
Supplement: Supplementary file 3 — Reporting Summary [file 44271_2025_208_MOESM3_ESM.pdf]

nature portfolio

Corresponding author(s): Professor Elizabeth Pellicano

Last updated by author(s): 01/20/2025

Reporting Summary

Nature Portfolio wishes to improve the reproducibility of the work that we publish. This form provides structure for consistency and transparency in reporting. For further information on Nature Portfolio policies, see our Editorial Policies and the Editorial Policy Checklist.

Statistics

For all statistical analyses, confirm that the following items are present in the figure legend, table legend, main text, or Methods section.

n/a	Confirmed	
☐	☒	The exact sample size (*n*) for each experimental group/condition, given as a discrete number and unit of measurement
☐	☒	A statement on whether measurements were taken from distinct samples or whether the same sample was measured repeatedly
☒	☐	The statistical test(s) used AND whether they are one- or two-sided<br>*Only common tests should be described solely by name; describe more complex techniques in the Methods section.*
☒	☐	A description of all covariates tested
☒	☐	A description of any assumptions or corrections, such as tests of normality and adjustment for multiple comparisons
☒	☐	A full description of the statistical parameters including central tendency (e.g. means) or other basic estimates (e.g. regression coefficient) AND variation (e.g. standard deviation) or associated estimates of uncertainty (e.g. confidence intervals)
☒	☐	For null hypothesis testing, the test statistic (e.g. F, t, r) with confidence intervals, effect sizes, degrees of freedom and P value noted<br>*Give P values as exact values whenever suitable.*
☒	☐	For Bayesian analysis, information on the choice of priors and Markov chain Monte Carlo settings
☒	☐	For hierarchical and complex designs, identification of the appropriate level for tests and full reporting of outcomes
☒	☐	Estimates of effect sizes (e.g. Cohen's *d*, Pearson's *r*), indicating how they were calculated

Our web collection on statistics for biologists contains articles on many of the points above.

Software and code

Policy information about availability of computer code

Data collection	Data collection did not require the use of specific software.
Data analysis	Data analysis was supported by NVivo version 14

For manuscripts utilizing custom algorithms or software that are central to the research but not yet described in published literature, software must be made available to editors and reviewers. We strongly encourage code deposition in a community repository (e.g. GitHub). See the Nature Portfolio guidelines for submitting code & software for further information.

Data

Policy information about availability of data

All manuscripts must include a data availability statement. This statement should provide the following information, where applicable:

- Accession codes, unique identifiers, or web links for publicly available datasets
- A description of any restrictions on data availability
- For clinical datasets or third party data, please ensure that the statement adheres to our policy

Raw qualitative data cannot be made open access due to ethical restrictions. De-identified qualitative data are available from the corresponding author upon request.

Human research participants

Policy information about studies involving human research participants and Sex and Gender in Research.

Reporting on sex and gender	We refer to participants' gender identity, which was self-reported. We did not collect information on sex because it was not relevant to our study aims, but our gender categories included transmen and transwomen (see Table 1).
Population characteristics	We report on participants' self-reported racial/ethnic background. We also report on their Autistic identity. We report on Autistic participants' diagnostic status and other co-occurring conditions.
Recruitment	Autistic participants were recruited through social media, and therefore represent a convenience sample.
Ethics oversight	Ethical approval for this study was granted by the Human Research Ethics Committee at Macquarie University (Project ID 6665).

Note that full information on the approval of the study protocol must also be provided in the manuscript.

Field-specific reporting

Please select the one below that is the best fit for your research. If you are not sure, read the appropriate sections before making your selection.

☐ Life sciences ☒ Behavioural & social sciences ☐ Ecological, evolutionary & environmental sciences

For a reference copy of the document with all sections, see nature.com/documents/nr-reporting-summary-flat.pdf

Behavioural & social sciences study design

All studies must disclose on these points even when the disclosure is negative.

Study description	This study analyses in-depth qualitative responses.
Research sample	95 Autistic participants from that study, including 16 young Autistic people and 79 Autistic adults
Sampling strategy	The sampling stratgy was self-selection as participants expressed interest by responding to an advert on social media.
Data collection	Data were collected through individual interviews via Zoom, telephone, email or live text.
Timing	May - June 2020
Data exclusions	The full sample included data from an additional 49 non-autistic participants, which have not been analysed here given that the focus is on Autistic people's experiences only.
Non-participation	No participants dropped out after starting the study.
Randomization	We did not randomise participants (given that this was a qualitative, not a quantitative study).

Reporting for specific materials, systems and methods

We require information from authors about some types of materials, experimental systems and methods used in many studies. Here, indicate whether each material, system or method listed is relevant to your study. If you are not sure if a list item applies to your research, read the appropriate section before selecting a response.

Materials & experimental systems

n/a	Involved in the study
☒	☐ Antibodies
☒	☐ Eukaryotic cell lines
☒	☐ Palaeontology and archaeology
☒	☐ Animals and other organisms
☒	☐ Clinical data
☒	☐ Dual use research of concern

Methods

n/a	Involved in the study
☒	☐ ChIP-seq
☒	☐ Flow cytometry
☒	☐ MRI-based neuroimaging
